# Psychobehavioral factors and family functioning in mucopolysaccharidosis: preliminary studies

**DOI:** 10.3389/fpubh.2024.1305878

**Published:** 2024-01-24

**Authors:** Daniel Almeida do Valle, Tiago dos Santos Bara, Vanessa Furlin, Mara Lúcia Schmitz Ferreira Santos, Mara L. Cordeiro

**Affiliations:** ^1^Faculdades Pequeno Príncipe, Curitiba, Brazil; ^2^Instituto de Pesquisa Pelé Pequeno Príncipe, Curitiba, Brazil; ^3^Department of Child Neurology Hospital Pequeno Príncipe, Curitiba, Brazil; ^4^Department of Psychiatry and Biological Behavioral Sciences, University of California, Los Angeles, Los Angeles, CA, United States

**Keywords:** mucopolysaccharidoses, family functioning, inherited metabolic diseases, cognitive function, psychobehavioral effects

## Abstract

**Introduction:**

Mucopolysaccharidoses (MPS) constitute a group of progressive and multisystemic inherited metabolic diseases that profoundly affect both the mental health of patients and the wellbeing of their families. This study aims to evaluate the impact of MPS on family functioning and related factors.

**Methods and results:**

Twenty-five patients with MPS, including types I (*n* = 4), II (*n* = 11), IIIB (*n* = 2), IVA (*n* = 3), and VI (*n* = 5), and their families participated in this study. The mean patient age was 13 years [standard deviation (SD): 7.7 years]. Behavioral and emotional problems were noted in 9.1% of all patients. While the type of MPS did not directly influence mental problems, the presence of neuronal involvement did (*p* = 0.006). Patients with MPS III exhibited difficulties primarily in emotional areas, conduct, hyperactivity, and peer problems. Importantly, both patients with MPS II and those with MPS III experienced a significant impact on communication [mean scores for communication domain: MPS II, 35.6 (SD: 24.3); MPS III, 35.0 (SD: 22.6)]; poorer communication was directly linked to worse adaptive behavior (*p* = 0.012), and worse adaptive behavior was associated with lower quality of life (*p* = 0.001). Quality of life and caregiver burden among family members did not significantly differ across MPS types; however, higher caregiver burden was negatively associated with quality of life (*p* = 0.002). Concerning family functioning, the most impacted domains included independence, intellectual/cultural orientation, activity/recreation, and expressiveness. Domain scores did not vary based on MPS type, treatment, or neurological involvement. Quality-of-life scores were positively associated with the cultural/intellectual domain score.

**Conclusion:**

The impacts of quality of life and family extend beyond clinical characteristics and MPS type, strongly influenced by patient cognition and communication, as well as type of family functioning, especially those with greater cultural/intellectual skills of their family members. A multidisciplinary approach addressing the broader needs of individuals with MPS becomes essential. Techniques aimed at improving communication, including prompt interventions such as speech therapy and augmentative and alternative communication strategies, can contribute to overall family functioning improvement.

## 1 Introduction

Mucopolysaccharidoses (MPS) are a group of rare inherited metabolic diseases (IMDs) caused by a lysosomal enzyme deficiency that affects the catabolism of glycosaminoglycans (GAGs). This deficiency causes accumulation of intracellular substances, leading to a complex cascade of events that lead to dysfunction of several cellular processes and pathways; these include an abnormal composition of membranes (impacting vesicle fusion and trafficking), impairment of autophagy, impairment of mitochondrial function, oxidative stress, and dysregulation of signaling pathways (1, 2). Depending on the deficient enzyme, MPS can be classified into the following 14 types: I, II, IIIA, IIIB, IIIC, IIID, IIIE (involving arylsulfatase G deficiency, encoded by the *ARSG* gene), IVA, IVB, VI, VII, IX, X, and the MPS-plus syndrome (MPSPS). MPSPS is caused by pathogenic or likely pathogenic variants in the *VPS33A* gene; although this gene codes for a lysosomal hydrolase, pathogenic or likely pathogenic variants in it result in a massive accumulation of GAGs (3–8). All MPS types are chronic, progressive, and multisystem diseases (4, 9).

MPS has an extremely variable prognosis, which is influenced by the MPS type, genetic variant, residual activity of the deficient enzyme, efficiency of GAG metabolism, age at onset, speed of disease progression, age at treatment initiation (enzyme replacement therapy or hematopoietic cell transplantation), socioeconomic status, and several other factors (10–12).

IMDs adversely affect the psychosocial wellbeing of parents (13). Furthermore, the severity and clinical manifestations of IMDs, including cognitive and motor impairment, are associated with the quality of life of caregivers (14). This could be attributed to the increased need for support among patients to perform activities of daily living. These added responsibilities can directly affect the health and wellbeing of the family, which disrupts work performance and social life (14).

Parents of patients diagnosed with MPS face various challenges arising from the multisystemic nature of the disease, which encompasses orthopedic, vision, and hearing issues; speech disorders; and cardiac problems (15). For patients, these issues extend beyond the physical aspect; even in milder cases, they may contribute to psychological problems and hinder appropriate societal adaptation. Some patients, despite having the capacity to work, may remain at home; conversely, some patients may face obstacles due to psychological challenges while attending school, making it difficult for them to form friendships (16).

Patients sometimes express fears of being scrutinized, harbor guilt concerning their parents, and grapple with anxieties about the future (including aspects such as forming friendships, getting married, bearing economic responsibilities, and having employment) (16). Even in attenuated forms of the condition, the psychological challenges faced by these patients and their family members can be profound; this is because owing to a better understanding of their own situation, these patients may experience a unique set of psychological complexities as compared to patients with the severe phenotype who have intellectual disabilities (16). Conversely, individuals with severe neurological impairment tend to grow increasingly reliant on care and often present with behavioral issues, such as hyperactivity, mouthing, unusual body movements, and inattention, which can be particularly pronounced in those with MPS III who are aged 2–9 years (17). The behaviors and psychological characteristics of these patients undergo significant changes, and the parents/caregivers experience extreme stress that directly affects their daily functioning. In light of this, the provision of psychological care to both patients and their family members or caregivers is indispensable (18).

Furthermore, since most MPS types involve autosomal recessive pattern of inheritance, parents may have two or three children with the disorder prior to the diagnosis of their first child (19). However, the psychosocial burden of MPS on parents in developing countries remain unclear.

Accordingly, this study aimed to evaluate the psychobehavioral effects of MPS on family functioning and related factors.

## 2 Materials and methods

### 2.1 Study design and population

This cross-sectional, observational, descriptive study was conducted in the Pequeno Príncipe Children’s Hospital and approved by our Ethics Committee (protocol number 47925921.5.0000.0097). All methods were performed in accordance with the guidelines and regulations of the Brazilian National Commission of Health (Commission of Ethics in Human Research-CEP/CONEP). The parents provided consent to the use of all data and images and for publication of this report.

We included participants with increased urinary glycosaminoglycans and laboratory-confirmed reduction in enzymatic activity; specifically, the enzymatic deficiency was defined as a reduction in enzymatic activity of <10% of the normal laboratory reference value.

Participants were further subgrouped according to MPS type, central nervous system involvement, and treatment performed [no treatment, enzyme replacement therapy (ERT), or hematopoietic cell transplantation].

### 2.2 Cognitive function

Estimated full-scale IQ was assessed using the Wechsler Preschool and Primary Scale of Intelligence-Revised (WPPSI-R) and Wechsler Abbreviated Scale of Intelligence (WASI); participants were administered the test that had been validated for their age.

The WPPSI-R was administered to children aged between 3 years 6 months and 5 years 11 months. The children received a four-subtest short version of the test comprising two subtests that assess perceptual–motor abilities. Raw scores obtained using the four subtests were converted into scaled scores (20).

The WASI was administered to children aged >6 years. It comprised four subtests (two verbal and two performance scales), which included the Vocabulary, Similarities, Block Design, and Matrix Reasoning subtests. Raw scores obtained using the four subtests were converted into scaled scores (21).

### 2.3 Children’s behavioral and emotional mental health

The Child Behavior Checklist (CBCL) was used to assess behavioral and emotional problems in children and Adult Self Report (ASR) was used for patients over 18 years of age during the previous 6 months (22, 23). It comprised 120 items, which were scored on a three-point scale: 0 (not true), 1 (somewhat or sometimes true), and 2 (very true or often true). It has excellent reliability and has been validated in the Brazilian population (24). The raw score was converted into T-scores by the Assessment Data Manager software and quantified within the following dimensions: Anxiety/Depression, Withdrawal, Somatic Complaints, Social Problems, Thinking Problems, Attention Problems, Rule-Breaking Behavior, Aggressive Behavior, Depressive Problems, Anxiety Problems, Somatic Problems, Attention Deficit/Hyperactivity Disorder, Oppositional Defiant Disorder, and Conduct Disorder. Additionally, the instrument can provide a Total Problem Score as well the Internalizing and Externalizing Problems scores (23). T-scores of ≤59, 60–64, and ≥ 65 indicate non-clinical symptoms, a risk for problem behaviors, and clinical symptoms, respectively (22, 23).

The Strength and Difficulties Questionnaire (SDQ) was used to assess problems related to mental health. The questionnaire comprises 25 items, including 10 items on abilities, 14 items on difficulties, and 1 neutral item. The instrument is divided into five subscales for assessing emotional symptoms (fears, excessive worries, sadness, and hopelessness), conduct problems (irritability, aggression, and antisocial behaviors such as lying), hyperactivity (restlessness, distraction, and inattention), problems with peer relationships (difficulties in relationships with other people, whether children or adults), and prosocial behavior (knowing how to cooperate, help, share). For each item, the individual could choose false (0 points), more or less true (1 point), and true (2 points). The score of each subscale ranges from 0 to 10, with a lower score indicating a better mental health status (25).

### 2.4 Adaptive behavior

Vineland Adaptive Behavior Scales (Vineland) was used to assess adaptative behavior. It involves a semi-structured interview using items scored as 0 (never performed), 1 (sometimes or partly performed), or 2 (behavior is usually or habitually performed). Normality was considered when score was 86 or higher (26, 27).

### 2.5 Family functioning

The Family Environment Scale (FES) is a self-reported 90-item scale for assessing family functioning across 10 different domains (28). We used the questionnaire version validated for Portuguese (29). It comprises five subscales, including Cohesion (commitment and family support); Expressiveness (direct communication of feelings), Conflict (express anger and conflict); Independence, Achievement Orientation, Intellectual Cultural Orientation, Active Recreational Orientation, Moral-Religious Emphasis, and Organization (maintenance of the family structure and organization); and Control (trust in rules and procedures to manage family life). The presence of problems is indicated by high scores on the Conflict and Control scales or low scores on the other scales (28). Table 1 presents the results grouped according to the type of family functioning.

**Table 1:** Classification according to the typology of the family environment (28).

Typology	Conditions
Independence orientation	Independence ≥69 and independence ≥ achievement/assertiveness
Achievement orientation/assertiveness	Achievement/assertiveness ≥60 and achievement/assertiveness ≥ intellectual/cultural AND moral / religiosity
Intellectual/cultural orientation	Intellectual/cultural ≥60
Moral and religious orientation	A—Moral/religious structure moral/religiosity ≥60 and moral/religiosity ≥ intellectual/cultural
	B—Moral/religious dysfunction moral/religiosity ≥60 and moral/religiosity ≥ intellectual/cultural and organization ≤50
Support guidance	Cohesion OR expressiveness OR both ≥60 and cohesion and expressiveness ≥ conflict
Conflict orientation	Conflict ≥60
Disorganization orientation	Organization ≤50

### 2.6 Caregiver burden

Caregiver burden was used to assess the version of the Zarit Burden Interview that has been translated and adapted to Portuguese (30, 31). The ZBI comprises 22 items rated on a 5-point Likert scale that ranges from 0 (never) to 4 (nearly always), with the total score ranging from 0 to 88. This tool allows assessment of objective and subjective burden among informal caregivers with respect to health, social life, personal life, finances, emotions, and relationship types.

### 2.7 Quality of life

We used the family impact module of the Pediatric Quality of Life Inventory™ to assess the impact of the disease and treatment on family functioning as well as the child’s adaptation to chronic diseases (32).

### 2.8 Coping techniques

The self-administered COPE Brief was used to investigate how individuals responded to stressful situations (33). It comprises 14 subscales for assessing coping techniques (self-distraction, active coping, denial, substance use, use of emotional support, use of instrumental support, behavioral disengagement, venting, positive reframing, planning, humor, acceptance, religion, and self-censorship).

### 2.9 Statistical analysis

Statistical analyses were performed using the Statistical Package for Social Sciences for Windows, version 22.0 (IBM Corp, Armonk, NY, United States). Descriptive analyses were used to obtain summary measures depending on the nature of variables. Further, inferential analysis was performed using study-relevant statistical tests (chi-squared and Fisher’s exact test). Continuous dependent variables were compared with categorical independent variables across all groups using the Kruskal–Wallis test. If the *p*-values obtained were significant, pairwise comparisons were performed to determine the differences between the groups. Statistical significance was set at *p* < 0.05.

## 3 Results

Among 56 patients diagnosed with MPS in our hospital (Curitiba, Paraná, Brazil), since 2005, 18 died before the commencement of neuropsychological assessments, eight refused to participate in the study, and five could not be contacted. Accordingly, 25 patients with MPS and their families were included, including four, 11, two, three, and five patients with MPS I, MPS II, MPS IIIB, MPS IVA, and MPS VI, respectively. The mean age was 13 years [standard deviation (SD): 7.7 years] (Table 2). ERT was performed in 50, 81.8, 100, and 100% of patients with MPS I, MPS II, MPS IVA, and MPS VI, respectively. Additionally, 50 and 18.2% of patients with MPS I and MPS II, respectively, underwent hematopoietic cell transplantation. None of the patients with MPS III received any specific treatment.

**Table 2:** Characteristics of the population studied according to the type of MPS^a^, psychometric characteristics, and family burden.

Variables		MPS I (*n* = 4)	MPS II (*n* = 11)	MPS III (*n* = 2)	MPS IVa (*n* = 3)	MPS VI (*n* = 5)	Total (*n* = 25)	*P*
Age (Years) Mean (SD)		10.7 (5.7)	13.1 (5.4)	9.5 (7.8)	19.0 (12.1)	14.0 (9.0)	13.3 (7.3)	0.802
Intellectual Quotient Mean (SD)		81.75 (33.7)	51.0 (17.4)	50 (14.1)	70.7 (12.9)	80.8 (36.4)	63.5 (26.1)	0.203
Capabilities and difficulties Mean (SD)	Total difficulties	10.0 (2.8)	11.5 (4.4)	25.0 (7.1)	5.7 (6.0)	8.3 (9.0)	11.2 (6.3)	0.081
	Emotional problems	2.0 (1.4)	2.3 (1.8)	6.5 (0.7)	1.7 (2.9)	2.3 (1.5)	2.5 (2.1)	0.227
	Conduct problems	1.5 (2.1)	1.7 (1.9)	3.0 (4.2)	0.3 (0.6)	1.3 (1.3)	1.5 (1.9)	0.823
	Hyperactivity	3.0 (4.2)	4.8 (3.1)	10.0 (0.0)	0.7 (1.2)	4.0 (0.8)	4.4 (3.3)	0.060
	Peer problems	3.5 (4.9)	2.7 (1.8)	5.5 (2.1)	3.0 (3.0)	0.75 (0.96)	2.7 (2.4)	0.201
	Prosocial	7.5 (3.5)	5.4 (3.4)	7.0 (0.0)	5.3 (5.0)	8.5 (1.9)	6.3 (3.3)	0.504
	Impact	4.0	3.2 (2.8)	0	2.5 (3.5)	0.25 (0.5)	2.2 (2.6)	0.168
Adaptive behavior Mean (SD)	Communication	89.5 (9.2)	35.6 (24.3)	36.0 (22.6)	94.7 (16.5)	72.8 (33.4)	58.0 (33.9)	0.037*
	Daily living skills	81.0 (14.1)	38.3 (27.1)	42.0 (31.1)	78.3 (20.0)	76.0 (44.5)	57.4 (34.4)	0.143
	Socialization	84.5 (2.1)	49.7 (26.0)	53.0 (15.6)	86.3 (26.1)	78.6 (36.3)	65.4 (29.8)	0.250
	Motor skills	82.5 (0.7)	42.1 (24.1)	45.5 (21.9)	82.7 (24.7)	76.0 (37.1)	60.1 (30.8)	0.387
Problems score Mean (SD)	Total	56.0 (5.7)	56.9 (6.9)	63.5 (17.7)	47.7 (7.6)	54.8 (6.7)	55.8 (8.2)	0.269
	Externalizing	55.0 (1.4)	49.5 (9.3)	56.0 (18.4)	43.3 (0.6)	51.5 (7.1)	50.1 (8.8)	0.215
	Internalizing	63.0 (9.9)	55.4 (5.2)	65.5 (10.6)	50.7 (9.1)	56.0 (8.5)	56.5 (7.6)	0.483
Quality of life Mean (SD)		65.4 (26.0)	69.4 (15.5)	47.1 (2.7)	67.7 (31.0)	77.4 (21.6)	68.6 (19.5)	0.353
Family burden Mean (SD)		27.5 (4.9)	20.7 (1.3)	24.5 (6.5)	14.0 (8.0)	24.4 (14.2)	21.6 (8.5)	0.289
Family environment Mean (SD)	Cohesion	56.3 (10.3)	57.3 (7.6)	48.0 (24.0)	54.0 (14.2)	53.2 (13.2)	55.0 (10.8)	0.983
	Expressiveness	46.7 (6.5)	51.1 (7.7)	43.5 (4.9)	50.7 (9.7)	48.2 (2.7)	49.2 (6.7)	0.605
	Conflict	49.3 (10.5)	48.0 (9.7)	52.0 (11.3)	40.3 (6.4)	45.2 (13.9)	46.9 (10.2)	0.582
	Independence	42.3 (9.2)	43.4 (7.4)	57.0 (5.7)	42.3 (4.6)	48.2 (12.1)	45.3 (8.8)	0.380
	Achievement	49.0 (3.5)	48.8 (8.5)	50.0 (8.6)	53.3 (12.5)	53.2 (11.5)	50.5 (8.6)	0.801
	Intellectual-cultural	48.7 (8.6)	45.6 (9.1)	41.5 (7.8)	50.3 (8.6)	47.8 (13.2)	46.7 (9.4)	0.799
	Active-recreational	48.0 (5.0)	50.3 (11.0)	53.0 (0.0)	46.7 (15.0)	51.2 (10.8)	50.0 (9.8)	0.805
	Moral-religious	59.3 (2.9)	55.0 (9.9)	56.0 (7.1)	59.3 (7.6)	56.0 (6.1)	56.4 (7.7)	0.908
	Organization	65.0 (3.5)	59.5 (4.7)	63.5 (7.8)	60.0 (10.8)	64.2 (2.7)	61.7 (5.5)	0.367
	Control	62.7 (6.4)	58.3 (10.0)	54.0 (7.1)	61.0 (3.5)	60.6 (8.7)	59.3 (8.2)	0.775

^a^MPS, mucopolysaccharidoses; SD, standard deviation. *Asterisks indicate statistical significance.

Table 2 summarizes the scores for the cognitive functioning and the strengths and difficulties subscales, as well as the total score of difficulties. Patients with MPS III exhibited difficulties predominantly in emotional areas, conduct, hyperactivity, and peer problems. The total score of difficulties was associated with the presence of neuronopathy (*p* = 0.001); however, no relationships were identified for the subscales.

The mean scores for the communication domain in adaptive behavior were below the cut-offs in patients with MPS II (35.6; SD: 24.3) and MPS III (35.0; SD: 22.6); significant differences were noted in the scores between patients with MPS II and those with MPS IV (*p* = 0.018). The total difficulty score was directly associated with impairment in the communication domain (*p* = 0.002). Both patients with high and very high scores in the conduct problems domain showed communicative impairment.

Additionally, 9.1, 4.5, and 9.1% of all patients exhibited behavioral or emotional problems, externalizing symptoms, and internalizing problems, respectively. These problems were more frequent (*p* = 0.017) in patients with neuronopathy (60.4, SD: 7.8) than in those without neurological involvement (51.9, SD: 6.4); however, they did not significantly differ according to age (*p* = 0.078) or time from diagnosis (*p* = 0.351).

The parents’ quality-of-life scores did not differ significantly across the different MPS types (*p* = 0.353) (Table 2). Regarding the impact on family functioning, the quality-of-life scores were positively associated with the cultural/intellectual domain score (*p* = 0.007); however, they were not associated with the other domains of family functioning or coping techniques used. Regarding adaptive behavior, the mean quality-of-life score was 63.14 (SD: 17.7) for parents of patients with low adaptive behavior and 94.3 (SD:6.9) for parents of patients with normal adaptive behavior (*p* = 0.001). There was no difference (*p* = 0.881) between the quality of life reported by family members according to the type of MPS (Table 2). In addition, self-reported quality-of-life scores were obtained from only eight participants. The scores did not significantly differ among patients with MPS I (72), MPS II (72.5, SD: 12.0), MPS VI (74.2; SD: 3.3), and MPS IVA (83.5, SD: 20.5).

Regarding family functioning and its domains, the adverse effects of MPS were observed in the domains of independence, intellectual/cultural, activity/recreation, and expressiveness (Table 2). However, no differences existed in any family functioning domains according to the MPS type, treatment type, or neurological involvement.

Families with an intellectual/cultural orientation had a better (*p* = 0.017) Problem-Focused Coping techniques (mean: 2.7; SD: 0.19) than families without an intellectual/cultural orientation (1.9, SD: 0.56).

For all patients, the mean caregiver stress score was 21.6; SD: 8.5. Moreover, 44, 40, and 4% of the family members reported no/mild, moderate, and severe burdens, respectively. Caregiver stress did not significantly differ according to the type of MPS (*p* = 0.289), treatment type (*p* = 0.489), or neurological involvement (*p* = 0.203). Caregiver burden was negatively associated with quality of life (*p* = 0.010).

The most reported coping techniques were Problem-Focused Coping techniques (average: 2.02; SD: 0.6), followed by Emotion-Focused Coping (1.68; SD: 0.43) and Avoidant Coping (0.26; SD: 0.26). The reported coping techniques did not significantly differ according to the type of MPS (*p* = 0.679, *p* = 0.209, and *p* = 0.534, respectively), type of treatment (*p* = 0.669, *p* = 0.991, and *p* = 0.762, respectively), or neurological involvement (*p* = 0.722, *p* = 0.107, and *p* = 0.418, respectively).

**Figure 1:**
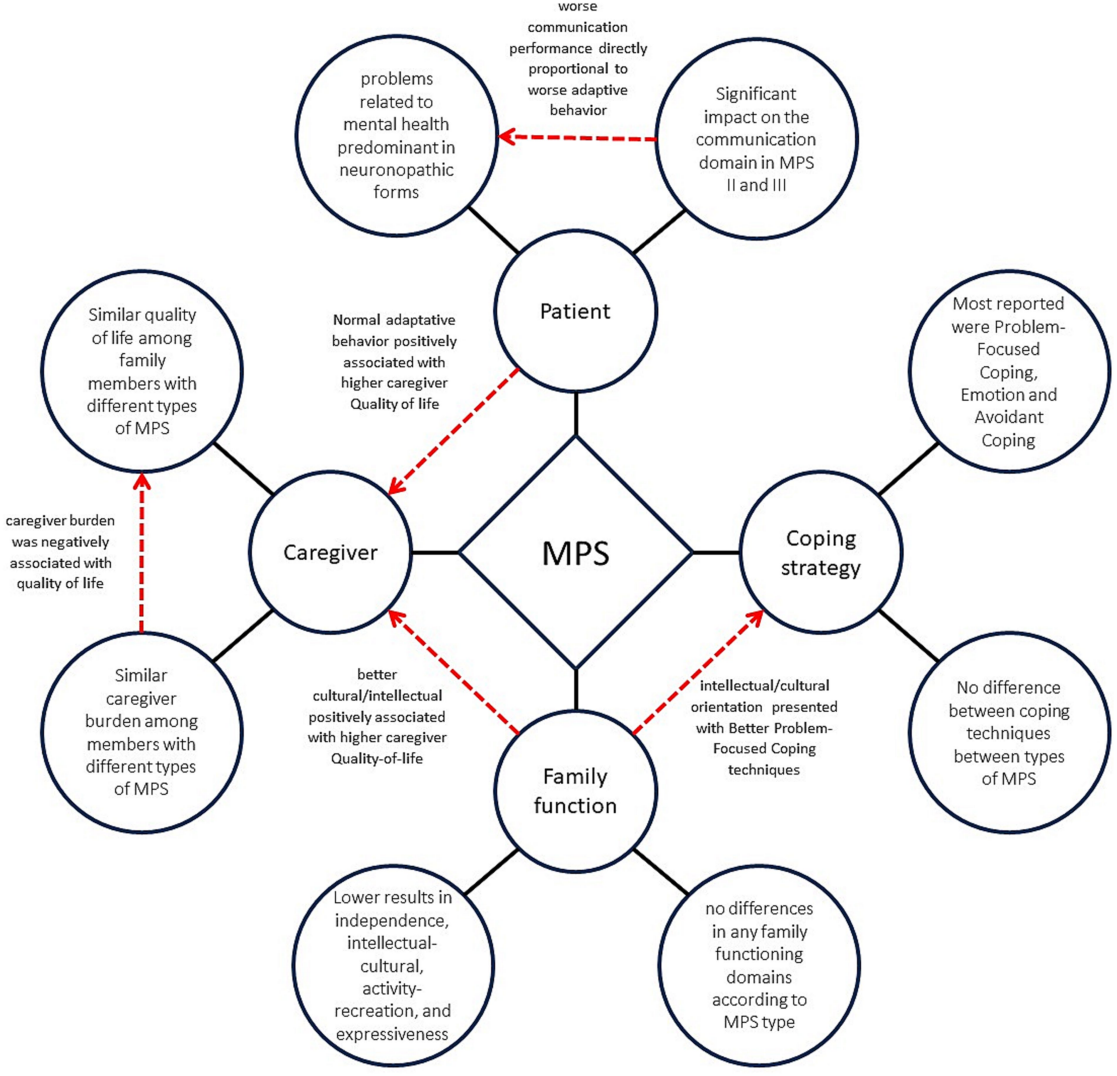
Psycho-behavioral factors and family functioning in mucopolysaccharidosis. The chart depicts the key elements of the mental health of individuals with MPS, the quality of life and stress experienced by their family members, and the family functioning and coping techniques that were identified in this study. It also depicts the correlations among these variables. Black lines represent the main factors identified, while red lines indicate the relationships among the different factors.

The main symptoms of the patient’s mental health, the family’s quality of life and functioning, and the coping techniques identified, as well as the relationships among them, are summarized in Figure 1.

## 4 Discussion

The neurocognitive impact of MPS widely varies from minor attention and executive function difficulties to severe intellectual disability (34). Similarly, we observed a wide range of cognitive impacts in patients with MPS II, ranging from mild effects to drastically low IQ scores, which indicates brain involvement and functional impairments despite them being considered as “non-neuronopathic” (34). Contrastingly, patients with MPS IV and MPS VI did not show progression of neurocognitive abnormalities, with most of them showing normal cognitive function (34). This is consistent with previous reports of relatively preserved cognitive functioning in these patients compared with those with other MPS types. Taken together, our findings emphasize the heterogeneity in the cognitive impacts of MPS and the need to perform individualized assessments and interventions.

Notably, we observed difficulties in cognitive functioning and adaptive behavior across several domains. Patients with MPS II and MPS III exhibited relatively lower scores in the communication domain than in the adaptive behavior domain. Indeed, speech, language, and communication impairments have been reported in patients with MPS, especially MPS II and III (34, 35). These impairments manifest as delayed language and speech development, limited vocabulary, speech absence, and overall impaired communication skills. Moreover, these communication deficits adversely affect their activities of daily life, especially expression of needs and desires (36, 37). Additionally, the total difficulty score was associated with adaptive performance in communication. The impact of these communication difficulties extends beyond the linguistic domain; instead, it limits social interactions, educational opportunities, and participation in various activities. Specifically, patients may experience frustration, social isolation, and difficulties in forming meaningful relationships (38). Over time, adaptative behavior may allow these children to cope with interpersonal issues even with persistent or worsening physical problems (36, 39). Therefore, adequate adaptive capacities can improve the psychosocial quality-of-life, which is consistent with the previous report by Shapiro et al. (39). Interventions targeting speech, language, and communication skills are crucial in supporting individuals with MPS to enhance their quality of life and promote their overall wellbeing (40). Specifically, prompt interventions, including speech therapy as well as augmentative and alternative communication strategies, can significantly improve communication outcomes and overall functioning (41). Additionally, multidisciplinary approaches that address the broader needs of individuals with MPS, including educational support and social skills training, can further enhance their communication abilities and optimize their participation in various aspects of life.

Our findings indicated a relationship between behavior disturbance and cognition in patients with MPS; specifically, IQ scores were negatively associated with a risk of behavioral issues. Consistent with previous reports, we found that scores for adaptive behaviors were lower in patients with MPS II and MPS III than those in general population, irrespective of treatment (36, 42). There has been insufficient research on behavioral, attentional, and executive function abnormalities in patients with MPS IV and MPS VI, which negatively affect the quality of life (34). However, our findings demonstrated the presence of emotional and peer problems in the MPS IVA group, albeit to a lesser extent than those in the MPS III group. Various behavioral problems have been reported in patients with MPS IVA, including anxiety/depression, attention difficulties, and somatic complaints (43). These findings demonstrated the need to address both cognitive and behavioral aspects when managing patients with MPS.

A Brazilian study reported a mean quality of life score of 48.1 in 11 mothers of children with MPS (14). In our study, except for MPS III, the other MPS groups had values higher than the aforementioned one, even in the presence of cognitive impairment. Contrastingly, they were lower than that reported by an Irish study (mean of 93.8) on patients with MPS, predominantly those with mild forms of the disease. The better quality of life observed in the Irish study could be attributed to a high level of social support (44). These inconsistencies in the reported impact of MPS on the quality of life may be attributed to several factors, including variability in the disease manifestations, treatment availability, and social support systems across different regions and healthcare systems. These factors can significantly affect the perception of quality of life by both individuals with MPS and their families. Furthermore, these inconsistencies can be attributed to the small sample sizes and potential cultural differences in the studied populations.

In our study, scores related to family functioning were lower in patients with MPS than in the healthy Brazilian population, especially in the domains of independence, intellectual/cultural, activity/recreation, and expressiveness (29). Children with MPS greatly rely on family members for assistance and support, which places a significant burden on the family and affect their overall functioning (36, 45, 46).

Furthermore, the responsibility to care give to children with MPS can adversely affect the parents’ working lives. Specifically, parents may be unemployed or forced to reduce their working hours to provide care for their children with MPS (13). Balancing caregiving responsibilities with work obligations can be challenging and cause financial strain and changes in career trajectories. These findings highlight the substantial impact of MPS on the family unit and the need for comprehensive support systems.

Inconsistent with previous reports, we observed no correlation between the severity of MPS and impact on families, which could be attributed to coping characteristics, including recognizing positive aspects of the caring process, reevaluating life goals, and receiving support from other affected families (13, 14, 36, 47).

The progressive and complex nature of MPS places significant demands on families and caregivers. The clinical manifestations of MPS can limit activities of daily living; moreover, the chronic and progressive disease nature can result in functional disability and a decrease in quality of life (39, 45, 46). Various MPS forms are related to behavioral problems that require coping strategies as well as time and physical presence from caregivers, which directly contribute to social isolation among families (48). This may explain the relatively greater impact in the independence domain. Generally, the severity of MPS symptoms is negatively associated with family functioning (36).

The caregiver burden reported by family members of children with MPS was found to be lower than that reported by family members of children with other chronic diseases (49) or Down syndrome (50). However, it was higher than that reported by family members of healthy Brazilian children (50). The caregiving responsibilities limit opportunities for leisure activities and social engagement. Moreover, these caregivers often experience parental stress, grief, feelings of loss, guilt, marital strain, and conflicts in their roles. Additionally, the chronicity of the disease contributes to family stress and imposes psychosocial demands on caregivers (36).

The caregiver burden is negatively associated with the quality of life for the patient, which indicates that the patient’s wellbeing influences the family dynamics. Notably, the wellbeing of the caregiver significantly influences the overall care provided to the child. Specifically, stress and burden levels among caregivers are negatively associated with their ability to provide optimal care and support to the child with MPS (51). Moreover, the caregiver burden is negatively associated with the perception of quality of life in pediatric patients (52). It is difficult to determine the causal relationship between caregiver stress and the child’s quality of life since these domains are interconnected and influenced by various factors. Accordingly, to elucidate this relationship, it is important to consider the multifaceted nature of MPS and its impact on the entire family unit.

In conclusion, our preliminary findings indicated that the impact of MPS on family functioning extends beyond physical aspects and encompasses social and emotional dimensions.

## Data availability statement

The raw data supporting the conclusions of this article will be made available by the authors, without undue reservation.

## Ethics statement

The studies involving humans were approved by the Pequeno Principe Hospital Ethics and Ethics Committee (protocol number 47925921.5.0000.0097). All methods were performed in accordance with the guidelines and regulations of the Brazilian National Commission of Health (Commission of Ethics in Human Research-CEP/CONEP). The studies were conducted in accordance with the local legislation and institutional requirements. Written informed consent for participation in this study was provided by the participants’ legal guardians/next of kin.

## Author contributions

DV: Conceptualization, Formal analysis, Investigation, Methodology, Validation, Writing – original draft, Writing – review & editing. TB: Formal analysis, Investigation, Software, Writing – review & editing. VF: Investigation, Writing – review & editing. MS: Investigation, Resources, Writing – review & editing. MC: Conceptualization, Data curation, Investigation, Methodology, Project administration, Resources, Supervision, Validation, Writing – review & editing, Writing – original draft.
